# The use of full-setting non-invasive ventilation in the home care of people with amyotrophic lateral sclerosis-motor neuron disease with end-stage respiratory muscle failure: a case series

**DOI:** 10.1186/1752-1947-6-42

**Published:** 2012-01-30

**Authors:** Eduardo L De Vito, Adrián A Suárez, Sergio G Monteiro

**Affiliations:** 1 Laboratorio Pulmonar de Enfermedades Neuromusculares, Instituto de Investigaciones Médicas Alfredo Lanari, Universidad de Buenos Aires, CONICET Combatientes de Malvinas 3150, CP 1427, Buenos Aires, Argentina

## Abstract

**Introduction:**

Little has been written about the use of non-invasive ventilation in the home care of amyotrophic lateral sclerosis-motor neuron disease patients with end-stage respiratory muscle failure. Nocturnal use of non-invasive ventilation has been reported to improve daytime blood gases but continuous non-invasive ventilation dependence has not been studied in this regard. There continues to be great variation by country, economics, physician interest and experience, local concepts of palliation, hospice requirements, and resources available for home care. We report a case series of home-based amyotrophic lateral sclerosis-motor neuron disease patients who refused tracheostomy and advanced non-invasive ventilation to full-setting, while maintaining normal alveolar ventilation and oxygenation in the course of the disease. Since this topic has been presented in only one center in the United States and nowhere else, it is appropriate to demonstrate that this can be done in other countries as well.

**Case presentation:**

We present here the cases of three Caucasian patients (a 51-year-old Caucasian man, a 45-year-old Caucasian woman and a 57-year-old Caucasian woman) with amyotrophic lateral sclerosis who developed continuous non-invasive ventilation dependence for 15 to 27 months without major complications and were able to maintain normal CO_2 _and pulse oxyhemoglobin saturation despite a non-measurable vital capacity. All patients were wheelchair-dependent and receiving riluzole 50 mg twice a day. Patient one developed mild-to-moderate bulbar-innervated muscle weakness. He refused tracheostomy but accepted percutaneous gastrostomy. Patient two had two lung infections, acute bronchitis and pneumonia, which were treated with antibiotics and cough assistance at home. Patient three had three chest infections (bronchitis and pneumonias) and asthmatic episodes treated with antibiotics, bronchodilators and cough assistance at home. All patients had normal speech while receiving positive pressure; they died suddenly and with normal oxygen saturation.

**Conclusions:**

Although warned that prognosis was poor as vital capacity diminished, our patients survived without invasive airway tubes and despite non-measurable vital capacity. No patient opted for tracheostomy. Our patients demonstrate the feasibility of resorting to full-setting non-invasive management to prolong survival, optimizing wellness and management at home, and the chance to die peacefully.

## Introduction

Amyotrophic lateral sclerosis-motor neuron disease (ALS-MND) is a rapidly progressive neurological disorder in which death is usually due to pulmonary complications and respiratory failure and survival time is two and a half to three years from onset of symptoms [1]. At least half of the patients die within 30 months of onset [2]. Tracheostomy and full-time ventilatory support are used to prolong survival in some centers; however, some people finally become cloistered up in those places (shut-in).

A close monitoring of the patient's physiological status and disease progression, and patient counseling on how to manage the options available are essential [3]. Because of its rapidly progressive nature, ALS-MND is considered appropriate for palliative care [4,5].

Recommendations about clinical respiratory (symptomatic) management are based on 'current evidence of best practice,' which largely comprises a conventionally accepted clinical opinion [6,7]. Symptomatic palliation is provided by supplemental oxygen and narcotic/sedative therapy which exacerbates hypoventilation and may hasten death, or by resorting to invasive airway cannulation and mechanical ventilation. Other options are to use nocturnal low-span bi-level positive pressure (BiPAP) ventilation to ameliorate symptoms until, with increasing respiratory muscle weakness this becomes inadequate and the patients develop acute respiratory failure (ARF), are intubated, undergo tracheostomy or die; or to use palliative drug therapy only until ARF develops and they die or need a tracheostomy [8,9].

Use of low-span nocturnal-only BiPAP has been reported to prolong the life of ALS patients by a few months [10-12]. However, one center has reported an eight-year survival in patients with ALS through the use of full-setting, continuous non-invasive ventilation (NIV) as needed [13] and has also reported similar benefits for patients with Duchenne muscular dystrophy [14,15]. The diagnosis of ALS was made on the basis of clinical and electromyographic findings, as defined by revised El Escorial criteria for diagnosing ALS [16].

We present three cases of home-based patients with ALS who refused tracheostomy and decided on full-setting NIV, continuous support for 15 to 27 months, maintaining normal alveolar ventilation and oxygenation in the process. It is important to point out that these patients needed NIV continuously at full ventilator settings. Unlike the low spans reported in the literature [10-12], all three patients required high span inspiratory positive airway pressure (16 to 20 cm H_2_O) and an expiratory positive airway pressure of 4 to 6 cm H_2_O, with a back-up rate of about 16 per minute.

## Case presentations

### Case 1

**Figure 1:**
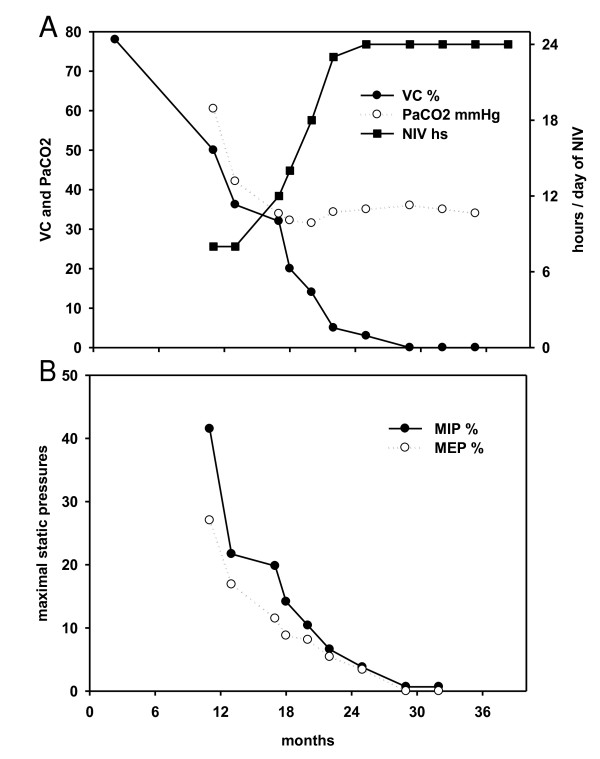
**Ratios regarding pulmonary function tests, hours using non-invasive ventilation (NIV) and months of evolution**. **Panel A **- Vital capacity (VC) in a percentage of the normal values, PaCO_2 _in mmHg and hours of NIV use. **Panel B **- Maximal static respiratory pressures at the mouth: maximal inspiratory pressure (MIP) and maximal expiratory pressure (MEP) are expressed as a percentage of the normal values. Tests show a worsening of the pulmonary function, maintaining normocapnia. The NIV duration was increased to full-time NIV for 16 months before death. During the last few months, VC was non-measurable, and MIP and MEP were zero or non-measurable.

A 51-year-old Caucasian man with ALS and an 11-month history of progressive generalized muscle weakness diagnosed five months after onset, was admitted to our hospital after a two-week history of dyspnea. He was wheelchair dependent. The patient had tachypnea and accessory muscle use. Lung auscultation was normal. Arterial blood gases when breathing ambient air were pH 7.42, PaO_2 _60.6 mmHg, PaCO_2 _60.5 mmHg, bicarbonate 39.2 mmol/L, and SatO_2 _91.3%, thus revealing chronic hypoventilation. Lung function (Vitalograph 2120 spirometer) demonstrated vital capacity (VC) 2.16 L (49% of normal), maximal inspiratory pressure (MIP) 40 cmH_2_O (38% of normal), maximal expiratory pressure (MEP) of 30 cmH_2_O (20% of normal), peak flow (PF) 100, peak cough flow (PCF) 110 L/minute. He began therapy with NIV via nasal interface during daytime naps and at night when sleeping. His general condition and dyspnea improved. Arterial blood gas obtained several days after starting NIV and prior to discharge was pH 7.39, PaO_2 _88 mmHg, PaCO_2 _41 mmHg, bicarbonate 24.8 mmol/L, SatO_2 _97%. His speech and video fluoroscopy of swallowing were normal and he was discharged home with an ALS Functional Rating Scale-Revised (ALSFRS-R) score of 17 (bulbar domain 9). As disease progressed, he developed mild-to-moderate bulbar-innervated muscle weakness. He accepted percutaneous gastrostomy. Twenty-six months after onset of muscle weakness he was 20-hour dependent on NIV. He died after having used NIV around-the-clock for 16 months. Clinical and functional follow-up results are shown in Figure 1.

### Case 2

A 45-year-old Caucasian woman with ALS was admitted to hospital because of dyspnea and morning headache. She presented with a 22-month history of progressive muscle weakness, beginning in her left hand. Four months after onset she was diagnosed with ALS. At presentation she had severe generalized muscle weakness and had been wheelchair dependent for the last month. Three months before, she had had pneumonia. Tachypnea and the use of accessory respiratory muscle were observed. Arterial blood gases when breathing ambient air were pH 7.41, PaO_2 _72 mmHg, PaCO_2 _50 mmHg, bicarbonate 31.6 mmol/L, and SatO_2 _95%; as in the previous case, chronic hypoventilation was observed. Her VC was 1.10 L (34% of normal), MIP 20 cmH_2_O (27% of normal), MEP 20 cmH_2_O (22%), PF 85 and PCF 100 L/min. Her speech was normal. Mild dysphagia and weak cough were observed. She began nasal NIV during daytime naps and overnight. Her general condition and dyspnea improved. Her arterial blood gases normalized at pH 7.40, PaO_2 _86 mmHg, PaCO_2 _40 mmHg, bicarbonate 24.7 mmol/L, and SatO_2 _97%. She was discharged home using nasal NIV with an ALSFRS-R score of 16 (bulbar domain 9). She became continuously NIV dependent within three months. She had two lung infections, acute bronchitis and pneumonia, which were treated with antibiotics and home cough assistance. Six months after the last infection she died at home after 15 months of continuous full-setting NIV dependence. For at least the last five months, her VC was non-measurable.

### Case 3

A 57-year-old Caucasian woman was admitted to hospital with supine dyspnea. She had a history of bronchial asthma and a 26-month history of progressive muscle weakness, beginning in her hands, during an intercurrent upper respiratory infection. She was diagnosed with ALS. Four pulmonary function tests performed over the past three years were normal. Three months before coming to us, her VC was 1.98 L (100% of normal). A week before that, she was unable to walk more than 200 meters because of her fatigue. She was tachypneic and was using accessory respiratory muscles. Orthopnea and abdominal paradoxing were present. Arterial blood gases when breathing ambient air were pH 7.42, PaO_2 _84 mmHg, PaCO_2 _38 mmHg, bicarbonate 24.6 mmol/L, and SatO_2 _97%. Her lung function measurements included VC 1.3 L (66% of normal) in the sitting position and 0.73 L supine, MIP 30 cmH_2_O (21% of normal), MEP 35 cmH_2_O (39% of normal), PF 120 and PCF 140 L/minute. Her speech was normal but mild dysphagia and weak cough were evident. She began nasal NIV during daytime naps and overnight and her dyspnea improved. She was discharged home with an ALSFRS-R score of 23 (bulbar domain 9). Twenty-three months after she had been 24-hour dependent on daytime nasal and nocturnal oronasal NIV, she had three chest infections (bronchitis and pneumonias) and asthmatic episodes treated with antibiotics, bronchodilators, and cough assistance at home. She died after 27 months of full-time, full-setting NIV at home. For at least the last eight months before she died, her VC was non-measurable.

## Discussion

There are some clinical aspects in common among all three patients. They had clinically definite ALS; consequently, they received riluzole 50 mg twice a day [5,6]. After discussing preferences and wishes, all patients refused tracheostomy. The main indication for NIV was the patients' self-reported symptoms (all three of them) and hypercapnia (the first two patients). All three died suddenly during the daytime, while talking to their loved ones. Perhaps they suffered a heart attack. The key points for the home management of these three patients were clinical vigilance, serial measurements of VC and coughing ability, cough assist with ambu bag, oxygen saturation overnight monitoring and advance planning.

As with tracheostomy ventilation (TV), full-time NIV may affect the patient's safety and comfort but NIV is invariably preferred by patients who have used both, and besides, noninvasive management results in fewer infections and hospitalizations [17-19]. Complications of both invasive and non-invasive management have been described [20,21]. The combination of nasal interface NIV during sleep and mouthpiece NIV when awake optimizes comfort but most ALS patients eventually develop too much lip weakness to use mouthpieces and, thus, must use nasal NIV around the clock. Patients still invariably prefer this to undergoing tracheostomy [17].

All three patients had been told that NIV would palliate symptoms and 'buy time' but that with a decreasing VC prognosis, this would result in their death. Unlike using the low BiPAP spans reported in the literature [10-12] that would buy little time, our patients were placed on high spans for up to full ventilatory support and/or respiratory muscle rest. An oximetry feedback protocol was used at home to screen for atelectasis, airway secretions, hypoventilation, and other pulmonary complications as described [22]. All patients' dyspnea and hypoventilation were completely relieved despite loss of all breathing ability (VC non-measurable) and they could only talk because of the pressure delivered by the BiPAP.

Although patients and physicians often consider NIV more desirable than invasive ventilatory support, with loss of all VC most clinicians continue to think that tracheostomy is necessary [23]. The cases reported in this paper support the supposition that this is not so [22]. However, with advancing bulbar-innervated muscle dysfunction such that SpO2 cannot be maintained at 95% or higher because of continuous saliva aspiration, tracheostomy does become necessary for survival [22]. Tracheostomy, however, can result in 20 years of institutionalization with loss of all motor abilities and sometimes communication skills ('shut-in'). As these cases demonstrate, the use of continuous NIV at settings sufficient for full ventilatory support may maximize the patient's chances of returning home and, eventually, dying there. NIV management, however, requires good patient and family involvement and realistic expectations [5].

There are now many papers on the importance of NIV for ALS-MND patients. Cazzolli and Oppenheimer reported on one patient who used continuous nasal NIV for two years [11]. Aboussouan *et al*. noted that those tolerating NIV, that is, those without severe bulbar-innervated muscle dysfunction, benefit the most. However, although bulbar dysfunction can decrease NIV tolerance, a trial is not contraindicated [12]. Bach described prolongation of survival in 36 patients with ALS by 14 to 17 months and, in some cases, for up to eight years by continuous NIV dependence, supplemented by mechanically assisted coughing, oximetry feedback as needed [13], and air stacking [24]. A decrease in the PF and MEP and in the difference between PCF and PF was observed, reflecting expiratory muscle weakness and bulbar impairment [25]. According to the ALSFRS-R scale, the bulbar involvement when patients were discharged home was mild to moderate. Despite the bulbar involvement, none of the patients performed a 'huffing' maneuver (which is common in patients with bulbar dysfunction) [26].

We think that survival was very dependent on bulbar function. With mild to moderate bulbar involvement, it was possible to maintain NIV with no major support or difficulties and using occasional cough assist. There were no major problems with the interfaces. According to the preferences of patients, they used different types of masks during the daytime and at night. In one case (patient two), on the occasion of testing the BiPAP apparatus with a DC car battery 12 V, the unit stopped working. It was thus necessary to connect the ambu bag in order to provide ventilation for three hours until replacement by the team.

There is no correlation of CO_2 _levels with a greater daily use of NIV. Symptomatic hypercapnia at any level should result in the introduction of NIV but once NIV has begun, its greater daily use does not correlate with a requirement for continuous use. Most patients who require continuous NIV have normal CO_2_, as also seen in our three patients. Figure 1 links daytime PaCO_2 _with the initiation of NIV and its greater daily use in the daytime. It can also be seen that PCO_2_ is normally maintained despite the lack of respiratory muscle strength; thus, VC is non-measurable.

We did not specifically assess the quality of life with special questionnaires. During the regular follow-up, patients' options were reviewed. Patients and their caregivers were glad to have chosen NIV into the home ventilation setting and would choose it again. Several studies indicate that NIV improved the symptoms of hypoventilation, thereby improving the quality of life and increasing survival of patients with ALS by several months. TV may increase survival more effectively but with a greater financial and care burden [10-12,27]. Bulbar symptoms partially account for intolerance of NIV, but should not interdict a trial of non-invasive positive-pressure ventilation [12]. In the Cazzolli survey, one hundred percent of the users indicated they were glad they chose NIV [11]. Of the 25 patients with tracheostomy who lived at home, 23 (92%) were satisfied with their quality of life, while seven (28%) of the other 25 who lived in a Skilled Nursing Care Facility, were not satisfied with their quality of life, and depression was a common issue [11].

A recent randomized controlled trial involving 92 ALS patients shows that NIV improves survival and quality of life [28]. In patients with severe bulbar impairment, NIV improves sleep-related symptoms, but is unlikely to confer a large survival advantage [28,29]. TV can prolong survival for many years, can be acceptable for some patients and caregivers and in these cases can improve patients' quality of life, although some patients become unable to communicate and remain in a shut-in state. However, home TV is costly and has a significant emotional and social impact on patients and caregivers. The advantages and drawbacks of TV have been described [30].

Unlike TV, NIV does not seem to be significantly detrimental to caregivers or patients who receive it [28]. Cazzolli shows that unlicensed paid attendants were employed by some families; and when properly trained, they provided excellent quality care [11]. Using NIV when possible is more cost-effective than TV due to lower costs for both equipment and caregivers. Home care of patients on long-term mechanical ventilation is challenging. The financial cost is great, with estimates ranging up to US$ 200,000/year including 24-hour care [31,32]. In Argentina, the annual cost of home care for invasive ventilation, including 24-hour care with skilled nursing, is about US$ 87,000, while for NIV it is about US$ 79,000. However, the cost for night nursing of NIV patients is about US$ 49,000 (case 3), whereas without nursing it can be reduced to about US$ 33,000 (cases 1 and 2).

## Conclusion

Little has been published about the use of NIV in the care of ALS-MND patients with end-stage respiratory muscle failure. There continue to be great variations by country, economics, physician's interest and experience, local concepts of palliation, hospice requirements, and resources available for home care. Our patients, however, demonstrate the feasibility of non-invasive management to prolong survival, optimize wellness and management at home, and optimize the chances to die peacefully.

## Consent

Written informed consent was obtained from the patients' next-of-kin for publication of this case report and any accompanying images. A copy of the written consent is available for review by the Editor-in-Chief of this journal.

## Competing interests

The authors declare that they have no competing interests.

## Authors' contributions

ELDV has made substantial contributions to the conception and design, and the follow-up of patients; he has been involved in drafting the manuscript and revising it from a critical standpoint due to its important intellectual content. AAS has made a substantial contribution to data acquisition, data analysis and interpretation, and the follow-up of patients; he has also revised the document from a critical standpoint due to its important intellectual contents. SGM dedicated his time to collecting data, following up of patients, revising the paper from a critical standpoint due to its important intellectual content. All authors read and approved the final manuscript.
